# Arabidopsis At5g39790 encodes a chloroplast-localized, carbohydrate-binding, coiled-coil domain-containing putative scaffold protein

**DOI:** 10.1186/1471-2229-8-120

**Published:** 2008-11-27

**Authors:** Elke M Lohmeier-Vogel, David Kerk, Mhairi Nimick, Susan Wrobel, Lori Vickerman, Douglas G Muench, Greg BG Moorhead

**Affiliations:** 1 Department of Biological Sciences and Alberta Ingenuity Center for Carbohydrate Science, University of Calgary, Calgary, Alberta, Canada, T2N 1N4

## Abstract

**Background:**

Starch accumulation and degradation in chloroplasts is accomplished by a suite of over 30 enzymes. Recent work has emphasized the importance of multi-protein complexes amongst the metabolic enzymes, and the action of associated non-enzymatic regulatory proteins. Arabidopsis At5g39790 encodes a protein of unknown function whose sequence was previously demonstrated to contain a putative carbohydrate-binding domain.

**Results:**

We here show that At5g39790 is chloroplast-localized, and binds starch, with a preference for amylose. The protein persists in starch binding under conditions of pH, redox and Mg^+2 ^concentrations characteristic of both the day and night chloroplast cycles. Bioinformatic analysis demonstrates a diurnal pattern of gene expression, with an accumulation of transcript during the light cycle and decline during the dark cycle. A corresponding diurnal pattern of change in protein levels in leaves is also observed. Sequence analysis shows that At5g39790 has a strongly-predicted coiled-coil domain. Similar analysis of the set of starch metabolic enzymes shows that several have strong to moderate coiled-coil potential. Gene expression analysis shows strongly correlated patterns of co-expression between At5g39790 and several starch metabolic enzymes.

**Conclusion:**

We propose that At5g39790 is a regulatory scaffold protein, persistently binding the starch granule, where it is positioned to interact by its coiled-coil domain with several potential starch metabolic enzyme binding-partners.

## Background

Research has shown us that the functions of proteins, including metabolic pathway enzymes, are highly regulated through compartmentalization, covalent modifications and specific protein-protein or protein-small molecule interactions. Most protein-protein interactions are mediated through specific domains that recognize other domains, motifs or modifications on other proteins [1]. This has lead to the compilation of a host of recognizable domains that bind other protein domains or other molecules, such as phosphoinositides, nucleic acids or carbohydrates. These are often easily detected through bioinformatic methods and provide clues about protein function. Among these domains are a collection of well characterized carbohydrate docking domains [2,3] one of which is present on the starch dephosphorylating enzyme SEX4. SEX4 belongs to the protein tyrosine phosphatase superfamily of protein phosphatases [4] and has been shown to be plastid localized, docks onto starch and is the functional equivalent of the *Homo sapiens *laforin phosphatase that dephosphorylates glycogen [5-8].

During our original analysis of the carbohydrate binding domain of SEX4 we noted several other Arabidopsis proteins with the equivalent carbohydrate docking domain, including At5g39790 [5]. At5g39790 is a small protein that has all the predicted amino acids necessary to bind starch. Here we have performed a biochemical and informatics analysis of At5g39790 and show that like SEX4, At5g39790 is highly conserved across plant species, is plastid localized, binds starch and is coordinately regulated at both the protein and message level along with other associated carbohydrate binding proteins. We have also noted that At5g39790 has a well-conserved predicted coiled-coil domain, like a number of the coordinately expressed starch metabolic enzymes and may function as protein interaction scaffold on the starch granule. This is consistent with recent genetic and biochemical data indicating that many of the starch metabolic enzymes interact physically [9,10].

## Results

### Bioinformatic analysis of At5g39790 predicts protein structural features, subcellular localization and gene expression patterns

**Figure 1:**
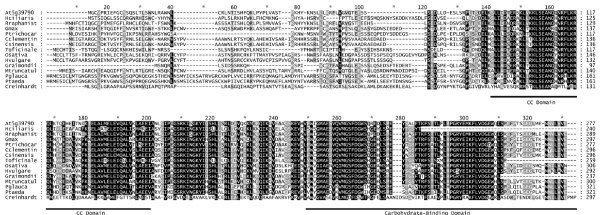
**Multiple sequence alignment of At5g39790 and homologues**. Homologue sequences were collected by database searching using the sequence of At5g39790 as the query, as detailed in Methods. A multiple sequence alignment was constructed from sub-alignments, and refined, as detailed in Methods. Species representation and the clones used to generate the sequences are as follows: At5g39790 (AY045645); Hciliaris (Helianthus ciliaris EL428795.1); Rraphanist (Raphanus raphanistrum EV525590 + EV529819); Gmax (Glycine max EH258682 + EH259354); Ptrichocar (Populus trichocarpa EF146538.1); Cclementin (Citrus clementina DY276384.1); Csinensis (Citrus sinensis CK933812 + CX302233); Toficinale (Taraxacum oficinale DY832182.1); Osativa (Oryza sativa CT832199.1); Hvulgare (Hordeum vulgare AK252768.1); Graimondii (Gossypium raimondii C0080363.1); Mtruncatul (Medicago truncatula DW015918.1 + BF003903); Pglauca (Picea glauca EX436035.1 + EX360120); Ptaeda (Pinus taeda DN462837.1 + CO362594.1); Creinhardt (Chlamydomonas reinhardtii XM_001694367.1). Structural domains were defined as detailed in Methods. Both the strongly predicted coiled-coil (CC) domain and the carbohydrate-binding domain (CBM) are indicated by underlined bars.

The amino acid sequence encoded by gene At5g39790 is presented along with the homologues we identified, in the multiple sequence alignment of Figure 1. Despite the presence of a few incomplete sequences, it is apparent by inspection that the carboxy-terminal region of the alignment is well conserved. A previous publication from our laboratory [5] showed the dual-specificity phosphatase SEX4, encoded by the gene At3g52180, contains a C-terminal carbohydrate-binding domain (CBM), which is shared with At5g39790 ([5,6]; Fig 1). This domain begins at residue 199 of At5g39790 (position 249 of the present alignment). Several residues were observed to be conserved in our previous work are also conserved in this alignment (G [position 262], W [position 267], G [position 296], K [position 301], G [position 306], W [position 308], and N [position 325].

The region immediately preceding the CBM is also well conserved in the alignment. We have analyzed the structural properties of this region as detailed in the Methods section. We found no evidence of characterized protein sequence domains. However, we found consistent evidence of a coiled-coil domain in all the sequences. This data is summarized in the Additional Table 1. There is a pattern of a strongly predicted region, followed by a more weakly predicted region. The stronger region extends from roughly residue 90 to 150 in the At5g39790 sequence (positions 140 to 200 in the alignment). The weaker region extends from roughly residue 160 to 195 in the At5g39790 sequence (positions 210 to 245 in the alignment).

It is evident that prior to about position 130 in the alignment, the N-terminal region is much less highly conserved. We have analyzed this set of sequences for the presence of chloroplast transit peptides, and the data are presented in Additional Table 2. It has been suggested previously [11] that sequences be judged positive for a chloroplast transit peptide if they achieve three out of four positive predictions in this test regimen. By that standard, sequence At5g39790 is predicted to be positive. By this criterion, one other sequence (that for *Taraxacum oficinale*) is also positive (4 of 4 tests). Three other sequences might be termed "possibly positive" on the basis of a positive result in 2 of the 4 tests (*Chlamydomonas reinhardtii*, *Glycine max*, and *Pinus taeda*). The remainder of the sequence set either has only one positive test (4 sequences) or none positive (6 sequences).

**Figure 2:**
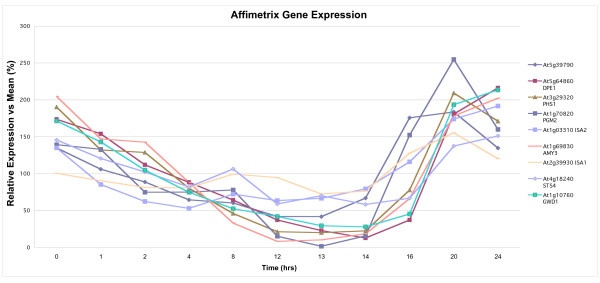
**Temporal pattern of gene expression for selected starch metabolic proteins**. The set of genes whose co-expression across the entire set of NASC microarray data is correlated with that of At5g39790 was obtained as detailed in Methods. Eight genes that encode plastid-localized starch metabolic proteins were noted where the Pearson correlation coefficient for co-expression exceeded r = 0.50. Diurnal gene expression data was then obtained from the NASC website as detailed in Methods, and placed into Microsoft Excel. Data were expressed as relative expression vs mean, using data from replicate experiments. Correlation coefficients were calculated comparing expression patterns of At5g39790 to each of the eight other genes, and the mean of this set of correlation coefficients is presented in the Figure. The individual correlation coefficients are as follows: At5g64860 (DPE1) 0.5937; At3g29320 (PHS1) 0.7696; At1g70820 (PGM2) 0.9231; At1g03310 (ISA2) 0.8061; At1g69830 (AMY3) 0.6890; At2g39930 (ISA1) 0.8534; At4g18240 (STS4) 0.5564; At1g10760 (GWD1) 0.6499. The first twelve hour period is the dark period, the second twelve hour period is the light period.

The presence of a well-conserved CBM and the presence of a predicted chloroplast transit peptide suggested to us the possibility that the protein product of gene At5g39790 participates in carbohydrate metabolism. We investigated this hypothesis further by examining patterns of correlated gene co-expression data as detailed in the Methods section. We examined a set of 33 genes known to be involved in starch metabolism (all except PGM2 (At1g70820) given in [12]). We found that eight of these have correlation coefficients greater than r = 0.50 (At2g39930 (ISA1) 0.6709; At5g64860 (DPE1) 0.6408; At3g29320 (PHS1) 0.6210; At1g70820 (PGM2) 0.6138; At4g18240 (STS4) 0.5914; At1g69830 (AMY3) 0.5699; At1g03310 (ISA2) 0.5630; At1g10760 (GWD1) 0.5429). We next examined the patterns of expression for At5g39790 under various experimental conditions at the Genevestigator site. It appeared that expression levels varied in a diurnal pattern. We have downloaded the original microarray data for the diurnal experiment of Smith et al. [12], and have compared the temporal expression patterns of At5g39790 with the set of eight starch metabolic enzyme genes cited above whose general co-expression pattern are most highly correlated as revealed at the Arabidopsis Co-expression Tool site. The results are shown in Figure 2. It is readily apparent that At5g39790 and these starch metabolic enzymes show a similar overall diurnal pattern, with expression declining during the first half of the experiment (the dark cycle), and rising to a maximum near the end of the experiment (during the light cycle). The correlation coefficients between the expression pattern of At5g39790 and each of these genes are as follows: At1g70820 (PGM2) 0.9231; At2g39930 (ISA1) 0.8534; At1g03310 (ISA2) 0.8061; At3g29320 (PHS1) 0.7696; At1g69830 (AMY3) 0.6890; At1g10760 (GWD1) 0.6499; At5g64860 (DPE1) 0.5927;At4g18240 (STS4) 0.5564. The mean correlation coefficient for this set of data is r = 0.730.

The presence of a strongly predicted coiled-coil (CC) domain in the amino acid sequence of At5g39790 (Fig 1) suggested to us the possibility of protein-protein interactions. We therefore screened the reference set of starch metabolic proteins for predictions of chloroplast transit peptides (which we expected to be positive) and CC domains. 15 of the 33 sequences proved to be negative for CC domains by all three tests. The other 18 had various CC regions predicted, at different strengths, in the different methods. The evidence for the subset of 7 proteins with the strongest CC predictions is presented in Additional Table 3 and the full set of predictions is summarized in Additional Table 4. There is at least moderate support by one prediction method, and reinforcement by a second method, for CC domains in five starch synthetic enzymes (At1g70820 (PGM2), At1g32900 (GBS1), At3g01180 (STS2), At1g11720 (STS3), At4g18240 (STS4)) and two starch degradative enzymes (At1g69830 (AMY3), At1g10760 (GWD1)). The evidence is most definitive for STS3 and STS4, where strong predictions are obtained by all three methods for at least one region each (there are also weaker predictions for a possible 2 additional areas in STS3). STS4 is especially notable in that the predicted coiled-coil region is exceptionally long (probably encompassing more than 200 residues). Of the 33 starch metabolic proteins set, 9 failed to achieve a consensus positive chloroplast transit peptide prediction (data summarized in Additional Table 5). All of these failures have been confirmed to be plastidic by experiment [13,14].

In order to provide context for the general gene co-expression results with At5g39790, we used the Arabidopsis Coexpression Data Mining Tools site to obtain the correlation coefficients for all-against-all comparisons of the 33 genes in the starch metabolic proteins set, over the full NASC microarray database. We set an arbitrary cutoff of r = 0.70 and above to define the strongest class of gene co-expression correlations. We found, as one would expect, a pattern of highly correlated co-expression between genes encoding starch synthetic enzymes, and also between genes encoding starch degradative enzymes. However, we also observed a number of instances of unexpected high correlation between starch synthetic and starch degradative enzymes. The correlated gene groupings are depicted in Additional Table 6. The numerical values for all the gene co-expression correlation comparisons are presented in Additional Table 7. The most remarkable standout in this collection of data is At4g18240 (STS4). It has strongly correlated co-expression with the largest number of other starch metabolic enzyme genes (ten in total), and strong correlations with the largest number of starch degradative enzyme genes (eight in total).

### Carbohydrate binding preference of At5g39790

**Figure 3:**
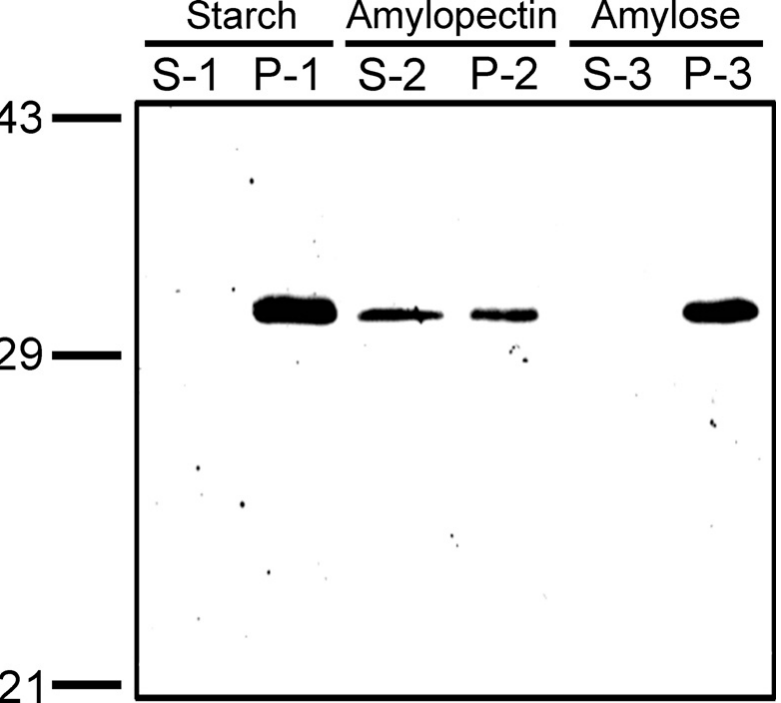
**Carbohydrate binding of At5g39790**. Purified At5g39790 was incubated with starch, amylopectin or amylose as in Methods. Western analysis of the supernatant (S) or pellet (P) fractions of starch (1), amylopectin (2) and amylose (3) associated protein are as indictaed. Samples (equal loading) were run on 12% SDS-PAGE gels, blotted and probed with antibodies generated against the protein.

In order to test that At5g39790 is a carbohydrate binding protein, as predicted by [5] (and Fig 1 in this study), a carbohydrate binding experiment was performed with At5g39790 cloned, expressed and purified without the putative chloroplast transit peptide (-CTP; see Materials and Methods). The protein was incubated with starch, amylopectin or amylose, and was observed in the pellet fractions with all three carbohydrates by Western blot analysis, as shown in Figure 3. These results confirm the prediction that this gene product is indeed a carbohydrate binding protein. The predicted molecular weight is close to 30,000, as expected due to the presence of the pRSET purification tag (see Materials and Methods). Although the protein bound all three carbohydrates, some unbound protein was observed in the supernatant of the amylopectin sample, indicating that At5g39790 has a preference for the longer α 1→4 linked glucose units found in amylose rather than the shorter regions found in α 1→6 branched amylopectin.

**Figure 4:**
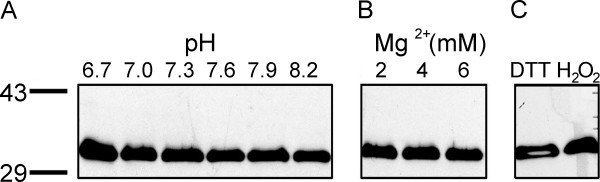
**Effect of pH, magnesium and redox state on starch binding of At5g39790**. Purified At5g39790 was incubated with starch as a function of pH in PBS as described in Methods. Samples were processed as in Fig. legend 3. Only the pellets are illustrated: (a) incubation of At5g39790 as a function of pH in phosphate buffered saline (PBS); (b) incubation of At5g39790 as a function of Mg^2+ ^concentration in PBS, pH 7.3) (the 0 mM Mg^2+ ^point is the same as in Fig 4a, lane 3); (c) incubation of At5g39790 under day (reducing [100 mM DTT], pH 8.0, 1 mM Mg^2+^) and night (oxidizing [30 mM H_2_O_2]_, pH 7.0, 1 mM Mg^2+^) stromal conditions.

The binding of At5g39790 to starch was also studied as a function pH, redox state and magnesium ion concentration. During the day the stromal pH is more alkaline, and redox conditions more reducing, than at night [15,16]. As well, stromal magnesium ion concentrations fluctuate between 1 mM during the day and 3 mM at night [17]. The results of this study, presented in Figure 4, show that At5g39790 does not dissociate from starch as a function of pH, Mg^2+ ^concentration, or redox state.

### Diurnal protein accumulation profile of At5g39790 is correlated with microarray mRNA transcript levels

**Figure 5:**
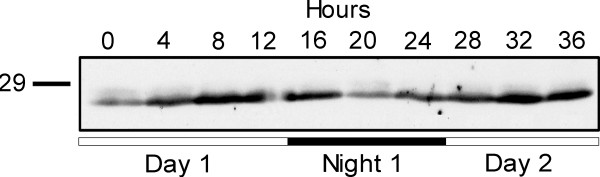
**Dirunal changes in At5g39790 protein in spinach leaf**. Spinach plants were grow in 12 hour cycle of light and dark and harvested at the times shown. Samples were processed, run on 12% SDS-PAGE, blotted and probed with antibodies generated against At5g39790. The open and solid bars below the blot indicate if samples were taken in the light (open bar) or dark (solid bar). The equivalent of 7.5 mg fresh weight leaf extract was analyzed for each sample.

The low natural abundance of At5g39790 in *A. thaliana *leaves made detection of signals on Western blots difficult, so the spinach (*S. oleracea*) results are presented here. Our ability to obtain a stronger signal in spinach is unclear and may simply reflect higher expression in spinach for some unknown reason. Results in Figure 5 show that protein levels increase during the day and decrease at night over the course of 36 hours when equal loading of fresh weigh leaf extracts were analyzed. Focusing on the data between time points 12–36 h shows a direct correlation between the protein level and the *A. thaliana *mRNA levels determined by microarray analysis ([12]; Figure 2 this study). Both fluctuate synchronously during day/night conditions. In a control Western blot experiment using only the secondary antibody, no protein was detected (results not shown).

Earlier research to monitor diurnal changes in starch metabolizing enzymes [12] was also performed using fresh weight leaf extracts for both mRNA analysis by microarray and transcript analysis by Western blotting. These researchers showed that many proteins involved in starch biosynthesis had protein levels that stayed constant even when their mRNA levels fluctuated diurnally (AMY3, GWD1 and DPE2, for instance). It was proposed that protein levels were under post-transcriptional control. An exception was granule-bound starch synthase, GBS1, a protein embedded in the starch matrix responsible for the synthesis of the amylose fraction of starch ([18,19]). Protein levels for GBS1 increased during the day, and decreased at night as the starch granule was degraded [12]. Our results with At5g39790 show a similar pattern, and this is not unexpected given the fact that dissociation from starch as a function of pH, Mg^2+ ^concentration, or redox state was not observed.

### Chloroplast localization of At5g39790

**Figure 6:**
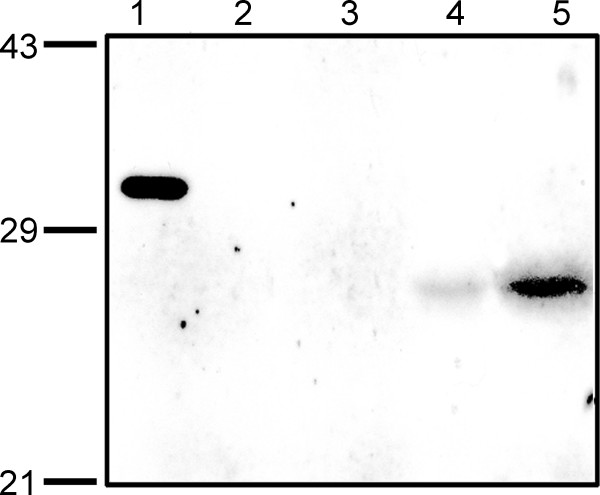
**Localization of At5g39790 homologue to *Spinaceao leracea *chloroplasts**. Spinach chloroplast and cytosolic fractions were probed with antibodies to At5g39790 (-CTP). Lane 1: At5g39790 (-CTP) control, 2 ng; lane 2: 1 μg cytosolic protein; lane 3: 2 μg cytosolic protein; lane 4: 1 μg chloroplast protein, Lane 5: 2 μg chloroplast protein. The difference in molecular weight of the native protein in lanes 4 and 5, compared to that in lane 1, is due to the presence of the N-terminal purification tag in our cloned protein (see Methods).

**Figure 7:**
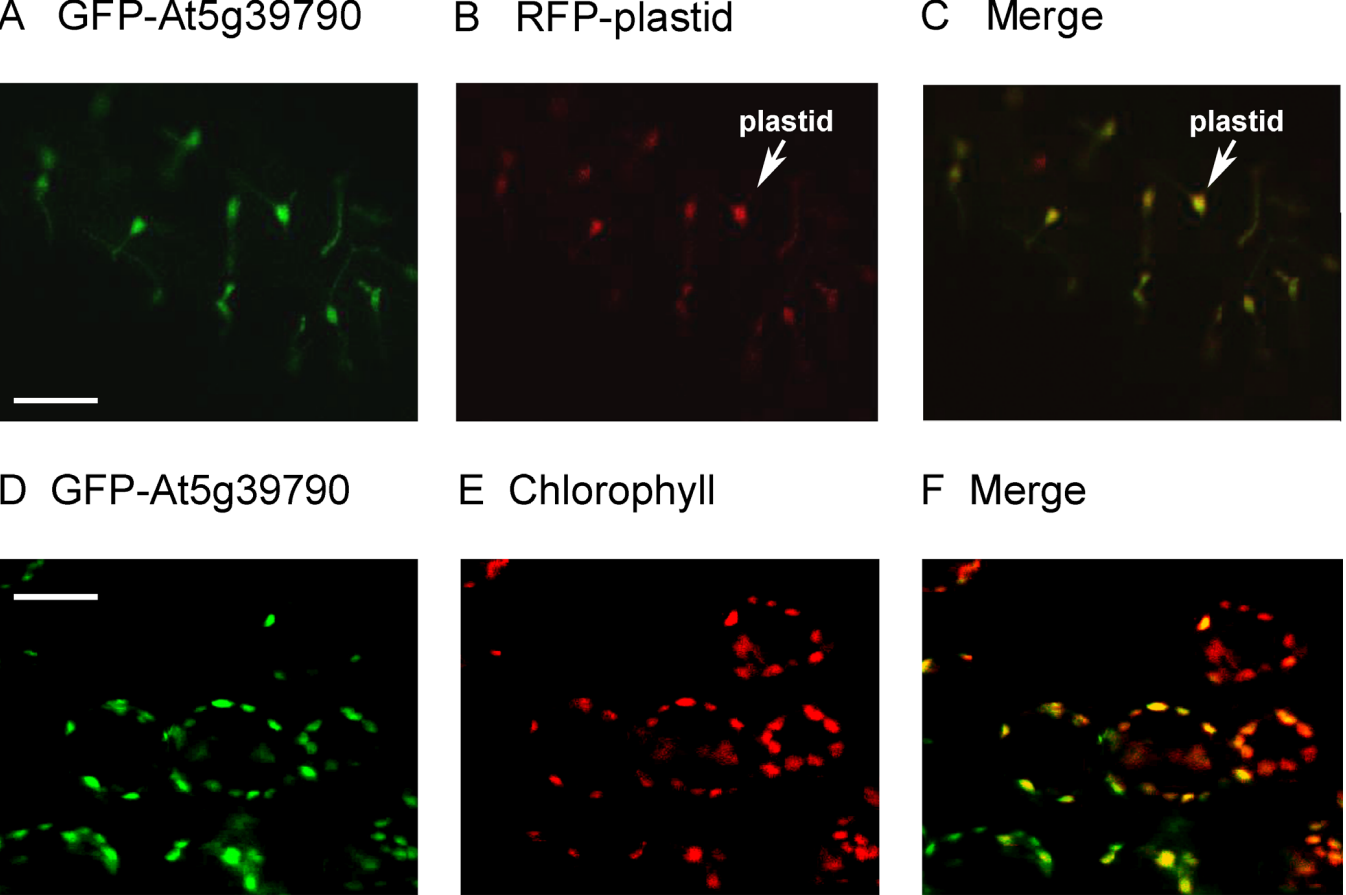
**Targeting of At5g39790 to plastids**. Onion (Allium aflatunense) epidermal cells were bombarded with 1.2 nm gold particles coated with the Agrobacterium tumefaciens pK7FWG2 plasmid containing At5g39790-GFP or the plastid control red fluorescent protein (RFP; [20]). Panel A: GFP targeting, panel B: RFP targeting. panel C: merging of panels A and B. The lower panels are a transgenic plant generated to express At5g39790-GFP. At5g39790-GFP fluorescence (panel D), chlorophyll fluorescence (panel E) and merged images D and E (panel F).

The data presented in Additional Table 2 of this study predicts that At5g39790 is chloroplast localized. To test this prediction we performed three experiments. In the first experiment chloroplasts were isolated from spinach instead of *A. thaliana *(for reasons mentioned in the preceding section) and probed with antibodies for the presence of At5g39790. Results in Figure 6 show that our protein is indeed localized in the chloroplast fraction of spinach isolates and absent in the cytosolic fraction. The second experiment utilized a C-terminal GFP fusion construct of At5g39790 that contained the putative chloroplast transit peptide (+CTP; see Methods). Plasmids containing the GFP fusion construct or the plastid targeted red fluorescent protein (RFP) control [20], were bombarded into onion epidermal cells. Figure 7 shows that after 12 hours the GFP-At5g39790 and RFP co-localize to the chloroplast (panels A-C). Thus the presence of the putative transit peptide indeed targets At5g39790 to this organelle. The third approach was to generate a transgenic *Arabidopsis thaliana *plant expressing At5g39790-GFP. This shows a clear localization of the enzyme to chloroplasts as evidenced by chlorophyll fluorescence (panels E-F).

## Discussion

We have shown by experiment that the protein encoded by *Arabidopsis *gene At5g39790 is chloroplast-localized, and that it binds starch. This is consistent with bioinformatic analysis of the amino acid sequence, which predicts a chloroplast transit peptide, and a well-conserved C-terminal carbohydrate-binding domain (present study, also [5]). Other structural analysis strongly predicts the presence of a coiled-coil domain. Such regions most often consist of a repeating heptad pattern of regularly spaced, alternating hydrophobic and hydrophilic residues, resulting in an alpha-helical supercoil between partner protein interaction surfaces [21,22]. Many examples have been characterized, including motor and other cytoskeletal proteins, chromosomal and membrane organizing proteins, and transcription factors. The presence of both a starch-binding domain and a coiled-coil domain in the structure of At5g39790 suggests the hypothesis that this is a scaffolding protein, whose structural properties would allow it to bind to the starch granule while also interacting with other proteins by way of its coiled-coil domain.

Analysis of gene expression patterns strongly supports the involvement of At5g39790 in a coordinately-regulated set of carbohydrate-metabolizing proteins. The correlation coefficients are substantial for general co-expression across a large number of microarray experiments between At5g39790 and several starch metabolic proteins. In particular, there is a clear diurnal pattern of expression which At5g39790 shares with a set of starch metabolic proteins. Smith et al [12] in their original presentation of this diurnal experiment microarray data commented in particular on the marked co-expression pattern of a group of starch degradative enzyme-encoding genes. We find that At5g39790 shares an evident pattern of co-expression with several of these degradative enzyme genes, but also with the gene for the synthetic enzyme STS4 (At4g18240). The role of STS4 has not been defined clearly, but is expected to function in starch synthesis.

Our structural analysis of a set of starch metabolic enzymes showed appreciable coiled-coil (CC) potential in several of their amino acid sequences. This was especially notable in the starch synthases. There is a body of previous work, both genetic and biochemical, showing the importance of multi-protein complexes in starch synthesis [23]. Recently, in wheat amyloplasts, homo-dimeric complexes between SBE isoforms have been observed, and hetero-multimeric complexes between SSI or SSII and SBE isoforms [9]. In *Zea mays *amyloplasts, a number of pair-wise interactions were demonstrated between starch synthetic enzymes. Of particular interest are large complexes involving SSII and SSIII. The latter was shown to interact with SSI in a region ("SSIIIHD") that shares similarity with a number of other SSIIIs from various plant species [10]. This region aligns with the portion of the *Arabidopsis *STS3 (At1g11720) sequence containing CC regions, and in fact we have found that the *Zea mays *sequence also contains CC potential in this region similar to the *Arabidopsis *protein (data not shown). Hence CC interactions should be considered likely candidates to explain at least some of the multiple protein-protein interactions known to be occurring in the starch synthetic enzyme system.

In our structural analysis, *Arabidopsis *STS4 (At4g18240) was distinctive in having the largest predicted CC region, probably extending more than 200 residues. This indicates a high potential to participate in multiple CC interactions with other proteins. This might indicate that STS4 participates in a large multi-protein assembly. Relatively little is known about the details of STS4 function. However, STS4 mutant plants were recently analyzed [24] and it appears that this enzyme controls the number of starch granules in plastids, perhaps by initiating starch granule priming. The authors suggest that this is likely to occur in a large protein complex. In the present study we observed a surprising number of instances of correlated gene co-expression between starch synthetic and starch degradative enzyme genes. This was most marked for At4g18240 (STS4), which has high correlations with a number of starch degradative enzyme genes. Correlations of this type might be expected if starch synthetic enzymes and starch degradative enzymes were working together in a multi-protein complex. It has recently been shown that a heteromeric complex of Stisa1 and Stisa2 isoforms is necessary for the initiation of starch granule synthesis in potato [25]. These authors concluded that it is likely that such a situation also occurs in *Arabidopsis*. Indeed, our gene expression data show a high correlation between the general expression patterns of At4g18240 (STS4) and At1g03310 (ISA2), with only a slightly weaker correlation with At2g39930 (ISA1). The presence of a number of other such correlations in our dataset might indicate that there are further, as yet undetected, interactions between starch synthetic and starch degradative enzymes. Arabidopsis STS4 would appear to be a promising target for further investigations in this area.

The combination of gene co-expression data, protein sequence structural analysis, and the results of the carbohydrate binding studies presented in this study strongly support the hypothesis that At5g39790 is a scaffold protein closely involved in starch metabolism (see below). The high degree of conservation of the carbohydrate-binding domain and coiled-coil regions of the sequences of the homologues collected from other plant species makes it very likely that they share the same function. However, sequence heterogeneity in the amino-terminal region of this protein set is observed, and chloroplast transit peptide predictions have provided mixed results.

Bioinformatic prediction of chloroplast transit peptides is a difficult process, since there is little conservation observed at the primary or secondary sequence level in actual transit peptides [11]. Indeed, in this study, 9 of 33 starch metabolic protein sequences in Arabidopsis failed to attain the recommended performance of three out of four positive test predictions. All of these sequences except one are known to be plastid-localized [13]. On a larger scale, the Zybailov study identified 916 proteins as plastid-localized with a high degree of confidence, but of these only 86% could be assigned chloroplast transit peptides by the TargetP technique. The plastid localization of the At5g39790 homologues, which currently cannot be assigned chloroplast transit peptides with confidence, will require experimental investigation.

Our Western blot and fluorescent imaging results agree with the bioinformatics predictions that At5g39790 localizes to the chloroplast. The predicted carbohydrate-binding domain of At5g39790 has been confirmed with the starch binding studies. Our data suggest that At5g39790 preferentially docks to amylose. Given the nature of starch it is unclear why we did not see complete binding to amylopectin given that starch is ~25% amylose. We can propose that there is simply adequate binding sites for the protein if 25% of the starch is amylose. It is possible that the branching of amylopectin actually hinders At5g39790 binding and reflects a unique feature of At5g39790 and this may play a role in defining its in vivo function. Of interest is that this protein does not dissociate from starch as a function of pH, Mg^2+ ^concentration, or redox state. As such it is highly probable that this protein binds to starch under both day and night conditions [16], and functions as a scaffolding protein for other proteins to bind to via the CC domain.

Not all starch binding proteins bind starch tightly all the time. The binding of the dual-specificity protein phosphatase SEX4 (DSP4, At3g52180) has been shown, *in vitro *to be regulated both by pH and redox potential [7]. SEX4 is found to bind starch during the day but not at night. In contrast, α-glucan water dikinase (GWD) binds at night but not during the day [26]. Even more interesting is the observation that granule-bound starch synthase (GBS1), a protein embedded in the starch matrix ([18,19]) has protein levels which fluctuate diurnally [12] in the same manner as At5g39790. GBS1 does not have a carbohydrate-binding domain, and based on similar diurnal protein levels and starch localization, it is tempting to speculate that GBS1may be anchored by At5g39790. Future studies utilizing biochemical and molecular genetics approaches will address this question.

## Conclusion

We have found that the CBM protein At5g39790 is expressed in a diurnal fashion at both the transcript and protein level. At5g39790 is conserved among plant species and outside of the CBM, the protein maintains a conserved coiled-coil domain that may act a as scaffold to bind other proteins. Gene expression analysis shows a pattern that correlates well with the expression other starch metabolic enzymes. The expressed protein readily docks starch and analysis with an antibody generated against At5g39790 shows the protein is localized to chloroplasts as is a GFP construct of the protein.

## Methods

### Homologue detection

Preliminary BLASTP database searches of databases utilizing the amino acid sequence of At5g39790 (NP_568573.2) as a query versus the protein datasets of completely sequenced plants and green algae ([4], Methods) produced a set of high-scoring hit sequences. Upon further examination, these proved to have poor sequence conservation in the amino terminal region. This generated scepticism about possible sequence assembly and annotation mistakes and prompted a more stringent search criterion, detailed below.

We decided it was critical to obtain amino acid sequence that came directly from cDNA clones with verifiably intact 5' termini. The amino acid sequence of At5g39790 (NP_568573.2) was then used as a query in TBLASTN searches of the NCBI "est-others" database, and the "nucleotides (nr)" database, using default parameters . The nucleotide sequences of high-scoring hits were retrieved, and subjected to six-frame translations . The sequences of 5' clones were sought, where the 5' untranslated region could be clearly identified by one or more stop codons. The first methionine residue encoded after this 5' UTR was taken to be the initiator methionine. It was also required that the translated sequence extended far enough into the C-terminus to include at least a part of a conserved carbohydrate binding module (CBM) known to be present in that region of the query sequence [5]. It became evident immediately that the reference NCBI amino acid sequence (and also that retrieved from the TAIR database) has four residues left out that are actually represented in the cDNA clone data ("LKTQ", res 119–122). Utilizing these criteria, a set of 14 sequences was obtained.

In some instances, full-length amino acid sequences were obtained. In other instances, the 5' cDNA clones did not supply a full-length sequence. In these instances, the translated amino acid sequence of the 5' clone was used as a query in another round of TBLASTN searches at NCBI, of the "est-other" and "nucleotide (nr)" databases. In this second round the sequences of 3' clones were sought, which shared a substantial area of overlap with the translated sequence of the 5' clone, and which also included the C-terminus of the amino acid sequence. The translated sequences of the 5' and 3' clones were then combined to produce a full-length sequence. This succeeded in generating a full-length sequence for 11 of the 14 sequences. For three of the sequences, no suitable 3' clone was available. Species of origin and details of clones utilized are given in the legend to Figure 1.

### Multiple sequence alignment

It was noted during the TBLASTN searches described above that high-scoring hit sequences fell into two groups: those sharing detectable similarity to At5g39790 close to the N terminus (e.g. similarity beginning from between residue 2 and residue 21 of the Arabidopsis query sequence); and those where similarity began later (e.g. between query residues 37 and 100). Sequences in each set were aligned using the program MUSCLE [27], with default parameters. Then the two resulting alignments were combined into a single alignment using the "profile alignment" option of ClustalX [28]. Finally, the resulting alignment was visualized using the program GeneDoc [29] then further improved in the amino-terminal region by visual inspection and sequence rearrangement.

### Chloroplast transit peptide prediction

Plant sequences were analysed for the possible presence of chloroplast transit peptides by the following methods: iPSORT ([30]; ); PCLR ([31]; ); PREDOTAR ([32]; ); and TARGETP ([33]; ). IPSORT utilizes amino acid properties and patterns in a succession of decision nodes to make predictions; PCLR analyses the abundance of different amino acid types, using principle-component analysis and logistic regression; while both PREDOTAR and TARGETP are artificial neural network methods.

### Coiled-coil protein domain analysis

The amino acid sequence of At5g39790 and its homologues were tested for coiled-coil (CC) domains by several methods. PairCoil2 ([34]; ) and PCOILS ([35]; ) utilize a Position Specific Scoring Matrix (PSSM) approach in combination with a fixed-length sliding window (28 or 21 residues). The former technique is improved by using pairwise residue probabilities, the latter by using profile-profile comparisons. Marcoil ([36]; ) dispenses with a sliding window approach, using instead Hidden Markov Models (HMMs). Marcoil reports predicted CC sequence regions under various threshold probabilities for a true positive result (e.g. 10%, 50%, or 90% threshold). PCOILS presents a graphical output with predicted CC regions depicted against a vertical axis representing the probability for a true positive result (e.g. 0.90). PairCoil2 presents both a graphical and numerical output. Here the default threshold for a region being predicted as a coiled-coil is that the probability of a false positive result be less than 0.025.

### Characterization of sequence domains

The amino acid sequence of At5g39790 and its homologues were tested for the presence of sequence domains using the NCBI Conserved Domain Database ([37]; ) and the Simple Modular Architecture Research Tool (SMART) ([38]; ).

### Microarray gene expression analysis

Correlated gene co-expression data and Affimetrix microarray data within the NASC data set [39] were analyzed. Probe identities were obtained from input Arabidopsis Genome Initiative gene numbers at the Arabidopsis Co expression Data Mining Tools Web site ([40,41]; ). Analysis of correlated probes was performed using the "Coexpression Analysis over Available Array Experiments" option. Tabulated correlation values (Pearson correlation coefficients [r]) were recorded. Calculation of the correlation coefficient values is explained at the web site.

### Diurnal gene expression data

Preliminary examination of patterns of Arabidopsis Affimetrix microarray gene expression data were conducted at the Genevestigator site ([42]; ), using the "Northern" option. It was noted that genes of interest appeared to have diurnal variation in their levels of expression. The NASCarray web site  was used to directly access the original microarray data. The search and retrieval features at this site were used to retrieve data from the experiment: "Gene expression and carbohydrate metabolism through the diurnal cycle" ([12]; NASCArrays Experiment Reference Number: NASCARRAYS-60). Numerical data were then placed into Microsoft Excel, where calculations were performed and graphs constructed.

### Cloning of the At5g39790 gene products

All reagents and enzymes were purchased from Invitrogen (Carlsbad, CA, USA) unless otherwise stated. The pda04554 plasmid containing the sequence for the At5g39790 gene was obtained from the Riken Biological Resource Center (Japan). The sequence corresponds exactly to that obtained within the NCBI protein data base (NP_568573; gi:42568201) and nucleotide data base (NM_123342.2; gi:42568200).

For protein expression studies we PCR-amplified the gene sequence without the first 44 amino acids of the putative chloroplast transit peptide (CTP) from the pda04554 plasmid. The forward primer contained an engineered *Bam*H1 restriction site (bold) ^5'^CGCGC**GGATCC**GCTTCTACTCGAAAACATTACAAC^3'^. The reverse primer contained an engineered *Eco*R1 site (bold): ^5'^CGCGCGAATTCCTATTCCACCACTAAAACATTGTT^3'^. Primers were synthesized by the University Core DNA Services facility (Calgary). The restriction digested PCR product was ligated with T_4 _DNA ligase into the pRSET A vector to produce a His-tagged fusion construct. The total expected molecular weight of the recombinant protein was ~30 kDa. The construct was sequenced at the University Core DNA Services facility (Calgary).

For *in vivo *expression studies the full At5g39790 gene sequence (inclusive of CTP) was engineered with primers to introduce attB1 and attB2 recombination sites (bold letters in primer sequence below) at the N and C terminal regions of the gene (normal case letters in primer sequence below), respectively. The forward primer sequence was: ^5'^**GGGGACAAGTTTGTACAAAAAAGCAGGCTTC**GAAGGAGATACAATCATGGGATGTGTACCC^3'^. The reverse primer sequence was: ^5'^**GGGGACCACTTTGTACAAGAAAGCTGGGTC**TTCCAACCACTAAAACATTGTTC^3'^. After PCR, the fusion product was recombined into an entry vector pDonor201, using the Gateway BP Clonase II enzyme mix. Plasmids were transformed into *E. coli *DH5α, selected for kanamycin resistance before being recombined into the Gateway vector pK7FWG2 [43] upstream from green fluorescent protein (GFP) using the LR Clonase II enzyme mix.

### Protein expression and purification

Expression of At5g39790 (-CTP) in *E. coli *gave rise to both inclusion body and soluble protein fractions. Optimal soluble protein production occurred in *E. coli *was obtained with the Tuner p*LacI *strain (Novagen) grown at 30°C. Transformed cells were grown to OD600 0.4 in 4L Luria Broth containing 35 mg/L chloramphenicol and 100 mg/L ampicillin, induced with 0.1 mM IPTG and allowed to express for 8 hours. Cells were cooled (with shaking) to 4°C, harvested by centrifugation, resuspended in 35 mL ice-cold lysis buffer (25 mM Tris pH 7.5, 150 mM NaCl, 30 mM imidazole, 0.5 mM PMSF, 0.5 mM benzamidine and 5 μ/mL leupeptin), flash frozen and stored at -80°C until further processing. Cell disruption and protein purification on a Ni-NTA Sepharose column was subsequently performed exactly according to [44] except that binding to the column occurred in the presence of 30 mM imidazole instead of 10 mM. This greatly reduced the binding of native *E. coli *proteins binding to the matrix. Purified At5g39790 (-CTP) was dialyzed against several changes of phosphate-buffered saline (PBS), pH 7.3, concentrated in a Centricon device (10,000 MWt cut-off) and stored at -80°C in 50% glycerol until used. From 4L culture ~2.4 mg soluble protein was obtained.

For higher amounts of protein required for the generation of antibodies, At5g39790 was purified from inclusion bodies. The pellet fraction from French-pressure disrupted cells was washed in lysis buffer containing 1% Triton X-100 and homogenized with a dounce homogenizer (20 strokes). The suspension was pelleted at 50,000 × g for 20 min at 4°C and the pellet resuspended in Ni-NTA binding (above) and processed as follows: The washed inclusion bodies were homogenized as above, sonicated in a water bath for 20 min and then placed into a 50 mL Falcon tube for end-over end mixing to allow protein unfolding to take place. This protocol was followed by a 30 min centrifugation step at 125,000 × g. The supernatant was then processed with Ni-NTA chromatography (as above), except that binding, washing and elution in the presence of 6 M Urea took place at room temperature. The eluted protein was sequentially dialyzed into PBS containing 4 M, 2 M, 1 M and no urea, and lyophilized. Protein purity may be observed in Additional File 8. A similar degree of purity is also observed with purified soluble At5g39790.

Rabbit primary antibodies to the purified At5g39790 (-CTP) were made using standard procedures [45]. The antibodies were subsequently affinity purified on an At5g39790 (-CTP) column. First 0.14 mg pure protein was coupled to 1 g CH-Sepharose (Amsersham), according to manufacturer's directions (> 95% coupling efficiency). Rabbit serum was incubated with the matrix with end over end mixing for 2 h at room temperature, washed with 20 mL 10 mM Tris, pH 7.5 and eluted with 15 mL 100 mM glycine, pH 1.8. The 1 mL fractions were captured in Eppendorf tubes that contained 200 μL of 1 M Tris pH 8.8 to immediately neutralize the antibodies. After dialysis into PBS, fractions were concentrated and stored at -80°C until required.

Primary antibody for Western blotting experiments was made up to 1 μg/mL concentration in Western blocking buffer containing 5% (w/v) skim milk powder, 2 mg/mL BSA and azide to 0.05% (w/v) final concentration. Goat anti-rabbit secondary antibodies (Pierce, 31460) were diluted 1:5000 in Western blocking buffer containing 5% (w/v) skim milk powder.

### Polysaccharide binding assays

Non-hydrolyzed starch (Sigma) and amylopectin (Fluka, St. Louis MO) were made up to 200 mg/mL concentration PBS, pH 7.3. Amylose (Type III Sigma) was made up as a 25 mg/mL stock solution. In order to remove small particulates, all polysaccharide stock solutions were centrifuged for 30 s at 500 rpm, the supernatant decanted and the volume brought up again with fresh PBS. Because the At39790 (-CTP) protein pelleted out at 14,000 rpm, the binding assays differed slightly from those previously published [5]. Binding between protein (1 μg/mL) and polysaccharide (10 mg/mL) in microfuge tubes took place for 1 h at room temperature with end over end mixing in PBS. The polysaccharides were centrifuged for 30s at 500 rpm and the supernatant carefully transferred to new microfuge tube. The polysaccharide pellets were subsequently washed 5 times, each time with resuspension in fresh PBS, gentle mixing, centrifugation and removal of supernatant. Pellets after the final wash were carefully transferred into a fresh microfuge tube and mixed with an equal volume of 2× SDS-PAGE cocktail. The first supernatant samples were diluted 1/5^th ^with 5× SDS-PAGE cocktail. All samples were processed for 5 min at 95°C, after which SDS-PAGE gels were run with 20 μL of the pellets and 25 μL of supernatants to obtain equal loading intensities.

### Diurnal cycling experiments

Three-week old *A. thaliana *plants and purchased young spinach plants (10–12 adult leaf stage) were placed under a double set of Gro-lights (Philips, F40T12/Plant + Aquarium), with exact day-night cycling for 12 hours each cycle for two days prior to the sampling. Leaves were then harvested at 4 h time intervals, flash frozen in liquid nitrogen (*l*N_2_) and stored in sealed containers at -80°C until processing.

Frozen leaves were ground in a *l*N_2 _pre-cooled mortar and pestle (with more coolant added as required to keep the particles frozen). Between 125–150 mg ground leaves were introduced into *l*N_2 _pre-cooled, pre-weighed microfuge tubes and weighed to three decimal points on an analytical balance. An exact amount (μL/μg) of 2× SDS-PAGE cocktail was added to the leaves and after brief vortexing the samples were placed on ice. After all the leaves had been processed, the samples were heated to 95°C for 5 min, vortexed vigorously for 30 s, heated for another 5 min and vortexed again. Processed samples were stored at -20°C until analyzed as Western blots.

### Chloroplast purification protocol

Spinach leaves were processed in homogenization buffer (300 M sorbitol, 50 mM Tris, pH 7.9, 2 mM EDTA and 1 mM MgCl_2_) in a Waring blender and strained through Miracloth 1R (Calbiochem). Filtrate was centrifuged at 1500 × g for 5 min, and the supernatant subsequently centrifuged at 400 × g for 20 min. The pellet was resuspended in homogenization buffer and layered onto a Percoll step gradient (5 mL 23%, 10 mL 30%, 5 mL 60%) for 20 min at 8000 × g. The intact chloroplast layer, identified by microscopy, was removed, transferred to SDS-PAGE cocktail, boiled for 10 min and stored at -20°C.

### At5g39790-GFP expression studies

For short term plant expression studies, purified pK7FWG2 plasmid with the At5g39790 insert was incubated with 1.2 nm gold particles that were subsequently bombarded into onion epidermal cells [46], along with the plastid control red fluorescent protein (RFP) [20] that is known to be targeted to (and expressed in) chloroplasts. After an overnight incubation on moistened filter paper in a petri dish, the living cells were imaged using epifluorescence microscopy (Leica, DMR, Germany). Images were captured using a Plan Fluotar 40 lens along with a cooled CCD camera (Retiga 1350 EX; QImaging, Burnaby, BC, Canada) and digitized with Velocity Software (Version 4.0, Improvision, Waltham, MA). Transgenic plants were generated by the floral dip method [47] utilizing At5g39790-pK7FWG2.

## List of Abbreviations

AMY3: α-amylase 3; APL1,2,3 or 4: ADP-Glc pyrophosphorylase (large subunits 1,2,3 or 4); APS1 or 2: ADP-Glc pyrophosphorylase (small subunit 1 or small subunit-like); BAM1,2,3 or 4: β-amylase 1,2,3 or 4 isozymes CTP: chloroplast transit peptide; DPE1 or 2: glucanotransferase isozyme [disproportionating enzyme], DPE1 is plastidal, DPE2 is cytosolic; GBS1: granule-bound starch synthase; GLT1 (glucose transporter) GWD1: glucan-water-dikinase 1; GWD3: glucan-water-dikinase-like 3 [phospholucan-water-dikinase]; ISA1 or 2: isoamylase 1 or 2 [starch debranching enzyme isoforms] starch synthesis, ISA3: isoamylase 1 or 2 [starch debranching enzyme isoforms] starch degradation; LDA1: starch debranching enzyme:limit dextrinase [pullulanase]; MEX1: maltose exporter; PGI1: phosphglucoisomerase; PGM1 or 2: phosphoglucomutase isoforms 1 or 2; PHS1: glucan phosphorylase [plastidal]; SBE3: starch branching enzyme III; SEX4: dual-specificity phosphatase, previously referred to as DSP4; SSI, II, III or IV: (starch sythase isoforms I, II, III or IV, elongation of amylopectin chains) STS1,2,3 or 4: starch synthase isoforms I, II, III or IV; TPTI: triose phosphate/Pi translocator;

## Authors' contributions

GBGM, conceived of the study andDK and EL-Vparticipated in its design. ELV, SW, MN, LV and DGM carried out the biochemical studies. DK carried out the bioinformatics study. ELV and DK drafted the manuscript.

## Supplementary Material

Additional file 1 **Supplemental Table S1**. Coiled-coil potential of At5g39790 and homologue sequences. Homologue sequences were collected by searching databases with the sequence of At5g39790 as detailed in Methods. Amino acid sequences were subjected to analysis of coiled-coil potential using the web servers for each of the specified methods, under default running parameters. The scoring characteristics of each test are detailed in Methods. Strength of the predictions for a given coiled-coil sequence region were arbitrarily categorized as "Strong", "Moderate" or "Weak" according to the following criteria. "Strong" (Marcoil, Threshold > 90%; PairCoil2, P < 0.025; PCOILS, W = 21 and 28 P > 0.90); "Moderate" (Marcoil, Threshold > 50%; PairCoil2, P < 0.05; PCOILS, W = 21 or 28 P > 0.50); "Weak" (Marcoil, Threshold > 10%; PairCoil2, P < 0.10; PCOILS, W = 21 or 28 P > 0.20). Predictions were characterized as negative (data not shown) under the following criteria: (Marcoil, Threshold < 10%; PairCoil2, P > 0.10; PCOILS, W = 21 and 28 P < 0.20). Click here for file

Additional file 2 **Supplemental Table S2**. Chloroplast transit peptide predictions of At5g39790 and homologues. Homologue sequences were collected by searching databases with the sequence of At5g39790 as detailed in Methods. Amino acid sequences were subjected to predictions of subcellular localization based on transit peptides, using the web servers for each of the specified methods, under default running parameters. a This method tests for transit peptides characteristic of localization to chloroplast, mitochondrion, or ER. It presents no single numerical estimate of prediction strength. b This technique tests only for the presence of chloroplast transit peptides. A score in excess of the threshold score of 0.42 is considered positive. c This method tests for transit peptides characteristic of localization to chloroplast, mitochondrion, or ER. The probability of the predicted transit peptide is given. d This method obtains scores for presence of a chloroplast, mitochondrial or ER signal peptide, or for some other cellular localization. The winning prediction is placed in a 'reliability class' (RC) according to the difference between the winning score and the next highest score. The classes, in descending order of reliability, are RC1–RC5. Click here for file

Additional file 3 **Supplemental Table S3 **Coiled-coil potential for selected starch metabolite proteins. A set of 33 genes encoding plastidial enzymes of starch metabolism was collected (32 of them given in Smith et al., 2004). Amino acid sequences were subjected to analysis of coiled-coil potential using the web servers for each of the specified methods, under default running parameters. The scoring characteristics of each test are detailed in Methods. The arbitrary classification of predicted coiled-coil sequence regions into "Strong", "Moderate" and "Weak" or "Negative" is detailed in the legend to Table 1. To facilitate comparison between sets of predictions for the various proteins, the complete set of coiled-coil predictions for each protein sequence was then arbitrarily assigned a "Relative Coiled-Coil Potential" according to the following criteria: 0 – Negative predictions by all three methods; 1 – Prediction for a given coiled-coil region from only one method; 2 – Mutually reinforcing predictions from more than one method; 3 – Moderate or Strong predictions with reinforcement in more than one method; 4 – Mutually reinforcing strong predictions from all three methods; 5 – Same as 4 but also exceptionally long CC domain predicted. Only data from sequences with a ranking of 3 or greater are shown in this Table. The gene naming abbreviations are as follows (all except PGM2 from Smith et al., 2004, Table 1 and Smith et al., 2005, Table 1): PGM2 (phosphoglucomutase, isoform2), GBS1 (granule-bound starch synthase), STS2 (starch synthase II), STS3 (starch synthase III), STS4 (starch synthase IV), AMY3 (α-amylase 3), GWD1 (glucan-water-dikinase 1). Click here for file

Additional file 4 **Supplemental Table S4**. Coiled-coil potential for full set of starch metabolic proteins. A set of 33 genes encoding plastidial enzymes of starch metabolism was collected (32 of them given in Smith et al., 2004). Amino acid sequences were subjected to analysis of coiled-coil potential using the web servers for each of the specified methods, under default running parameters. The scoring characteristics of each test are detailed in Methods. The arbitrary classification of predicted coiled-coil sequence regions into "Strong", "Moderate" and "Weak" or "Negative" is detailed in the legend to Table 1. To facilitate comparison between sets of predictions for the various proteins, the complete set of coiled-coil predictions for each protein sequence was then arbitrarily assigned a "Relative Coiled-Coil Potential" according to the following criteria: 0 – Negative predictions by all three methods; 1 – Prediction for a given coiled-coil region from only one method; 2 – Mutually reinforcing predictions from more than one method; 3 – Moderate or Strong predictions with reinforcement in more than one method; 4 – Mutually reinforcing strong predictions from all three methods; 5 – Same as 4 but also exceptionally long CC domain predicted. Starch synthetic enzyme genes are highlighted in pale green. Starch degradative enzyme genes are highlighted in pale orange. The gene naming abbreviations are as detailed in Additional File 6. Click here for file

Additional file 5 **Supplemental Table S5**. Chloroplast transit peptide predictions for starch metabolic proteins. A set of 33 genes encoding plastidial enzymes of starch metabolism was collected (32 of them given in Smith et al., 2004). Amino acid sequences were subjected to predictions of subcellular localization based on transit peptides, using the web servers for each of the specified methods, under default running parameters. The gene naming abbreviations are as detailed in Additional File 6. a This method tests for transit peptides characteristic of localization to chloroplast, mitochondrion, or ER. It presents no single numerical estimate of prediction strength. b This technique tests only for the presence of chloroplast transit peptides. A score in excess of the threshold score of 0.42 is considered positive. c This method tests for transit peptides characteristic of localization to chloroplast, mitochondrion, or ER. The probability of the predicted transit peptide is given. d This method obtains scores for presence of a chloroplast, mitochondrial or ER signal peptide, or for some other cellular localization. The winning prediction is placed in a 'reliability class' (RC) according to the difference between the winning score and the next highest score. The classes, in descending order of reliability, are RC1–RC5. Click here for file

Additional file 6 **Supplemental Table S6**. Sets of starch metabolic enzyme genes with strong general co-expression correlation patterns. A set of 33 genes encoding plastidial enzymes of starch metabolism was collected (32 of them given in Smith et al., 2004). The Arabidopsis Coexpression Data Mining Tools site was accessed as detailed in Methods. Pearson correlation coefficients (r) expressing quantitatively the degree of gene co-expression observed over the entire NASC microarray dataset were obtained for pairwise all-against-all gene comparisons. An arbitrary standard of r greater than or equal to 0.70 was used to define the class of strongest gene co-expression correlations. This Table presents non-redundant groups of genes whose mutual comparisons produce correlation coefficients which meet this standard. (The set of all comparisons, with the numerical data, is presented as Supplemental Table 2). The gene naming abbreviations are as follows (all except PGM2 from Smith et al., 2004, Table 1 and Smith et al., 2005, Table 1): PGM1 (phosphoglucomutase, isoform1), APL1 (ADP-Glc pyrophosphorylase: large subunit 1), APS1 (ADP-Glc pyrophosphorylase: small subunit), GBS1 (granule-bound starch synthase), STS1 (starch synthase I), STS3 (starch synthase III), STS4 (starch synthase IV), SBE3 (starch branching enzyme III), SBE2 (starch branching enzyme II), ISA1 (isoamylase 1 [starch debranching enzyme]), ISA2 (isoamylase 2 [starch debranching enzyme]), AMY3 (α-amylase 3), GWD1 (glucan-water-dikinase 1), GWD3 (glucan water dikinase-like 3 [phosphoglucan-water-dikinase]), DPE1(glucanotransferase [disproportionating enzyme]), PHS1 (glucan phosphorylase [plastidial]), ISA3 (isoamylase 3 [starch debranching enzyme]), BAM2 (β-mylase 2), LDA1 (starch debranching enzyme:limit dextrinase [pullulanase]), MEX1 (maltose exporter). For convenience of comparison, gene names are grouped as "Starch Synthetic Enzymes" or "Starch Degradative Enzymes". Since ISA1 and ISA2 homologues in potato have been shown to be essential for starch synthesis (Bustos et el., 2004) they are here considered to be starch synthetic enzymes. Click here for file

Additional file 7 **Supplemental Table S7**. Gene co-expression correlations for At5g39790 and starch metabolic protein genes. A set of 33 genes encoding plastidial enzymes of starch metabolism was collected (32 of them given in Smith et al., 2004). The Arabidopsis Coexpression Data Mining Tools site was accessed as detailed in Methods. Pearson correlation coefficients (r) expressing quantitatively the degree of gene co-expression observed over the entire NASC microarray dataset were obtained for pairwise all-against-all gene comparisons, and are presented in this Table. Starch synthetic enzyme genes are highlighted in pale green. Starch degradative enzyme genes are highlighted in pale orange. At5g39790 is highlighted in pale purple. Gene co-expression correlations for At5g39790 and starch metabolic protein genes which exceed r = 0.50 are highlighted in pale blue. Gene co-expression correlations for pairs of starch metabolic protein genes which exceed r = 0.70 are highlighted in dark blue. The gene naming abbreviations are as detailed in Additional File 6. Click here for file

Additional file 8 **Supplemental Figure S1**. Purification of At5g39790 (-CTP) used for the preparation of antibodies in rabbit. Purification of At5g39790 for antibody preparation: Lane 1, Low MWt standards; lane 2, 3 μg French press supernatant; lane 3, 3 μg of French press pellet (50,000 × g); lane 4, 3 μg of urea supernatant; lane 5, 3 μg of Urea pellet (125,000 × g); lane 6, 3 μL Ni-NTA flow-through; lane 7, 30 μL column wash; lane 8, 3 μg column eluant. Click here for file
