# Supplementary material for: Arabidopsis At5g39790 encodes a chloroplast-localized, carbohydrate-binding, coiled-coil domain-containing putative scaffold protein
Source: BMC Plant Biol. 2008 Nov 27;8:120. doi: 10.1186/1471-2229-8-120 (PMC2653042; doi:10.1186/1471-2229-8-120)
Supplement: Additional file 1 — Supplemental Table S1. Coiled-coil potential of At5g39790 and homologue sequences. Homologue sequences were collected by searching databases with the sequence of At5g39790 as detailed in Methods. Amino acid sequences were subjected to analysis of coiled-coil potential using the web servers for each of the specified methods, under default running parameters. The scoring characteristics of each test are detailed in Methods. Strength of the predictions for a given coiled-coil sequence region were arbitrarily categorized as "Strong", "Moderate" or "Weak" according to the following criteria. "Strong" (Marcoil, Threshold > 90%; PairCoil2, P < 0.025; PCOILS, W = 21 and 28 P > 0.90); "Moderate" (Marcoil, Threshold > 50%; PairCoil2, P < 0.05; PCOILS, W = 21 or 28 P > 0.50); "Weak" (Marcoil, Threshold > 10%; PairCoil2, P < 0.10; PCOILS, W = 21 or 28 P > 0.20). Predictions were characterized as negative (data not shown) under the following criteria: (Marcoil, Threshold < 10%; PairCoil2, P > 0.10; PCOILS, W = 21 and 28 P < 0.20). [file 1471-2229-8-120-S1.pdf]

Additional Table 1: Coiled-Coil Potential of At5g39790 and Homologue Sequences

Sequence	Marcoil	PairCoil2	PCOILS
At5g39790	Strong(105-142,Th:90%)<br>Weak(165-191,Th:10%)	Strong(90-150)[P=0.011]<br>Moderate(160-195)[P=0.03]	Strong(110-150)[P=0.95]<br>Weak(160-190)[P=0.30]
Gossypium raimondii C0080363.1	Strong(78-123,Th:90%)<br>Moderate(154-170,Th:50%)	Strong(60-120)[P=0.011]<br>Weak(150-175)[P=0.068]	Strong(60-130)[P=1.0]<br>Moderate(150-175)[P=0.75]
Oryza sativa CT832199.1	Strong(127-174,Th:90%)<br>Weak(200-222,Th:10%)	Moderate(140-170)[P=0.02]<br>Weak(200-225)[P=0.056]	Strong(120-180)[P=1.0]<br>Weak(200-225)[P=0.30]
Hordeum vulgare AK252768.1	Strong(112-158,Th:90%)<br>Neg	Moderate(100-140)[P=0.03]<br>Weak(175-210)[P=0.094]	Strong(110-160)[P=1.0]<br>Weak(180-210)[P=0.25]
Chlamydomonas reinhardtii XM_001694367.1	Strong(118-142,Th:90%)<br>Moderate(174-200,Th:50%)	Strong(110-140)[P=0.008]<br>Moderate(175-210)[P=0.034]	Strong(120-150)[P=1.0]<br>Strong(170-200)[P=1.0]
Taraxacum oficinale DY832182.1	Strong(98-145,Th:90%)<br>Moderate(174-190,Th:50%)	Strong(90-140)[P=0.017]<br>Weak(160-200)[P=0.074]	Strong(90-150)[P=0.95-1.0]<br>Weak(160-190)[P=0.45]
Medicago truncatula DW015918.1	Strong(123-171,Th:90%)<br>Weak(196-213,Th:10%)	Strong(110-170)[P=0.015]<br>Weak(190-220)[P=0.095]	Strong(110-170)[P=1.0]<br>Weak(190-220)[P=0.35]
Raphanus raphanistrum EV525590	Strong(112-160,Th:90%)<br>Moderate(191-202,Th:50%)	Strong(100-160)[P=0.004]<br>Weak(180-220)[P=0.052]	Strong(100-170)[P=1.0]<br>Weak(180-210)[P=0.50]
Citrus clementina DY276384.1	Strong(118-165,Th:90%)<br>Moderate(194-205,Th:50%)	Strong(100-160)[P=0.009]<br>Neg	Strong(110-170)[P=1.0]<br>Weak(185-210)[P=0.50]
Populus trichocarpa EF146538.1	Strong(95-143,Th:90%)<br>Moderate(171-195,Th:50%)	Strong(90-150)[P=0.005]<br>Moderate(165-195)[P=0.028]	Strong(90-150)[P=1.0]<br>Moderate(160-195)[P=0.65]
Citrus sinensis CK933812	Strong(118-165,Th:90%)<br>Moderate(194-205,Th:50%)	Strong(100-160)[P=0.009]<br>Neg	Strong(100-170)[P=1.0]<br>Weak(185-220)[P=0.50]
Glycine max EH258682	Strong(121-168,Th:90%)<br>Moderate(192-212,Th:50%)	Strong(110-170)[P=0.006]<br>Moderate(185-220)[P=0.043]	Strong(120-180)[P=1.0]<br>Moderate(185-220)[P=0.65]
Helianthus ciliaris EL428795.1	Strong(106-152,Th:90%)<br>Moderate(182-198,Th:50%)	Moderate(130-160)[P=0.026]<br>Weak(180-210)[P=0.053]	Strong(100-150)[P=0.90-1.0]<br>Weak(180-205)[P=0.45]
Pinus taeda DN462837.1	Strong(144-190,Th:90%)<br>Weak(218-232,Th:10%)	Strong(140-200)[P=0.004]<br>Neg	Strong(135-200)[P=1.0]<br>Weak(215-240)[P=0.45]
Picea glauca EX436035.1	Strong(144-187,Th:90%)<br>Neg	Strong(140-200)[P=0.005]<br>Neg	Strong(140-200)[P=1.0]<br>Weak(215-235)[P=0.25]
