# Supplementary material for: Arabidopsis At5g39790 encodes a chloroplast-localized, carbohydrate-binding, coiled-coil domain-containing putative scaffold protein
Source: BMC Plant Biol. 2008 Nov 27;8:120. doi: 10.1186/1471-2229-8-120 (PMC2653042; doi:10.1186/1471-2229-8-120)
Supplement: Additional file 2 — Supplemental Table S2. Chloroplast transit peptide predictions of At5g39790 and homologues. Homologue sequences were collected by searching databases with the sequence of At5g39790 as detailed in Methods. Amino acid sequences were subjected to predictions of subcellular localization based on transit peptides, using the web servers for each of the specified methods, under default running parameters. a This method tests for transit peptides characteristic of localization to chloroplast, mitochondrion, or ER. It presents no single numerical estimate of prediction strength. b This technique tests only for the presence of chloroplast transit peptides. A score in excess of the threshold score of 0.42 is considered positive. c This method tests for transit peptides characteristic of localization to chloroplast, mitochondrion, or ER. The probability of the predicted transit peptide is given. d This method obtains scores for presence of a chloroplast, mitochondrial or ER signal peptide, or for some other cellular localization. The winning prediction is placed in a 'reliability class' (RC) according to the difference between the winning score and the next highest score. The classes, in descending order of reliability, are RC1–RC5. [file 1471-2229-8-120-S2.pdf]

Additional Table 2: Chloroplast Transit Peptide Predictions for At5g39790 and Homologues

**Sequences**	**iPSORT** a	**PCLR** b	**Predotar** c	**TargetP**	**Consensus Prediction**
At5g39790	Chloro	Pos(0.55)	Plastid(0.55)	Mito RC5 d	Chloro(3/4)
Gossypium raimondii C0080363.1	Mito	Neg(0.061)	None(0.99)	Mito RC3	Not chloro
Oryza sativa CT832199.1	None	Neg(0.329)	None(0.90)	None RC5	Not chloro
Hordeum vulgare AK252768.1	None	Neg(0.255)	None(0.93)	None RC3	Not chloro
Chlamydomonas reinhardtii XM_001694367.1	Mito	Pos(0.875)	Possibly mito(0.37)	Chloro RC4	Possibly chloro (2/4)
Taraxacum oficinale DY832182.1	Chloro	Pos(0.609)	Possibly plastid(0.21)	Chloro RC4	Chloro (4/4)
Medicago truncatula DW015918.1	None	Neg(0.129)	None(0.96)	Chloro RC3	Not chloro
Raphanus raphanistrum EV525590	Chlor	Neg(0.190)	None(0.85)	None RC5	Not chloro
Citrus clementina DY276384.1	None	Neg(0.133)	None(0.98)	None RC3	Not chloro
Populus trichocarpa EF146538.1	None	Neg(0.180)	None(0.94)	None RC4	Not chloro
Citrus sinensis CK933812	None	Neg(0.133)	None(0.98)	None RC3	Not chloro
Glycine max EH258682	None	Neg(0.344)	Possibly plastid(0.21)	Chloro RC3	Possibly chloro (2/4)
Helianthus ciliaris EL428795.1	Mito	Pos(0.474)	None(0.93)	Mito RC2	Not chloro
Pinus taeda DN462837.1	Mito	Pos(0.866)	None(0.93)	Chloro RC5	Possibly chloro (2/4)
Picea glauca EX436035.1	Mito	Pos(0.536)	None(0.97)	None RC4	Not chloro
