# Supplementary material for: Arabidopsis At5g39790 encodes a chloroplast-localized, carbohydrate-binding, coiled-coil domain-containing putative scaffold protein
Source: BMC Plant Biol. 2008 Nov 27;8:120. doi: 10.1186/1471-2229-8-120 (PMC2653042; doi:10.1186/1471-2229-8-120)
Supplement: Additional file 3 — Supplemental Table S3 Coiled-coil potential for selected starch metabolite proteins. A set of 33 genes encoding plastidial enzymes of starch metabolism was collected (32 of them given in Smith et al., 2004). Amino acid sequences were subjected to analysis of coiled-coil potential using the web servers for each of the specified methods, under default running parameters. The scoring characteristics of each test are detailed in Methods. The arbitrary classification of predicted coiled-coil sequence regions into "Strong", "Moderate" and "Weak" or "Negative" is detailed in the legend to Table 1. To facilitate comparison between sets of predictions for the various proteins, the complete set of coiled-coil predictions for each protein sequence was then arbitrarily assigned a "Relative Coiled-Coil Potential" according to the following criteria: 0 – Negative predictions by all three methods; 1 – Prediction for a given coiled-coil region from only one method; 2 – Mutually reinforcing predictions from more than one method; 3 – Moderate or Strong predictions with reinforcement in more than one method; 4 – Mutually reinforcing strong predictions from all three methods; 5 – Same as 4 but also exceptionally long CC domain predicted. Only data from sequences with a ranking of 3 or greater are shown in this Table. The gene naming abbreviations are as follows (all except PGM2 from Smith et al., 2004, Table 1 and Smith et al., 2005, Table 1): PGM2 (phosphoglucomutase, isoform2), GBS1 (granule-bound starch synthase), STS2 (starch synthase II), STS3 (starch synthase III), STS4 (starch synthase IV), AMY3 (α-amylase 3), GWD1 (glucan-water-dikinase 1). [file 1471-2229-8-120-S3.pdf]

Additional Table 3: Coiled-Coil Potential for Selected Starch Metabolic Proteins

**Sequence**	**Marcoil**	**PairCoil2**	**PCOILS**	**Relative CC Potential**
AT1G70820	Weak(61-73,Th:10%)	Moderate(50-75)[P=0.047]	Neg	3
PGM2	Weak(453-498,Th:10%)	Neg	Strong(440-480)[P=0.90]	
At1g32900	Weak(438-453:Th10%)	Moderate(400-450)[P=0.048]	Weak(425-450)[P=0.50]	3
GBS1				
At3g01180	Moderate(74-113,Th:50%)	Moderate(75-100)[P=0.027]	Weak(75-100)[P=0.35]	3
STS2				
At1g11720	Moderate(83-107,Th:50%)	Weak(75-125)[P=0.051]	Weak(100-140)[P=0.45]	4
STS3	Strong(239-281,Th:90%)	Strong(225-300)[P=0.003]	Strong(225-300)[P=0.80-0.95]	
	Moderate(425-452,Th:50%)	Moderate(425-475)[P=0.028]	Weak(425-475)[P=0.40]	
At4g18240	Strong(192-260,Th:90%)	Strong(200-400)[P=0.00134]	Strong(190-450)[P=1.0]	5
STS4	Strong(280-306,Th:90%)			
	Strong(316-344,Th:90%)			
	Strong(363-374,Th:90%)			
	Strong(380-458,Th:90%)			
At1g69830	Strong(424-455,Th:90%)	Moderate(425-450)[P=0.026]	Neg	3
AMY3				
At1g10760	Moderate(235-253,Th:50%]	Weak(225-250)[P=0.053]	Weak(225-250)[P=0.45]	3
GWD1	Moderate(497-507,Th:50%]	Weak(480-510)[P=0.063]	Weak(500-525)[P=0.25]	
	Weak(561-575,Th:10%)	Neg	Weak(550-600)[P=0.25]	
	Neg	Weak(1125-1150)[P=0.09]	Neg	
