# Supplementary material for: Arabidopsis At5g39790 encodes a chloroplast-localized, carbohydrate-binding, coiled-coil domain-containing putative scaffold protein
Source: BMC Plant Biol. 2008 Nov 27;8:120. doi: 10.1186/1471-2229-8-120 (PMC2653042; doi:10.1186/1471-2229-8-120)
Supplement: Additional file 5 — Supplemental Table S5. Chloroplast transit peptide predictions for starch metabolic proteins. A set of 33 genes encoding plastidial enzymes of starch metabolism was collected (32 of them given in Smith et al., 2004). Amino acid sequences were subjected to predictions of subcellular localization based on transit peptides, using the web servers for each of the specified methods, under default running parameters. The gene naming abbreviations are as detailed in Additional File 6. a This method tests for transit peptides characteristic of localization to chloroplast, mitochondrion, or ER. It presents no single numerical estimate of prediction strength. b This technique tests only for the presence of chloroplast transit peptides. A score in excess of the threshold score of 0.42 is considered positive. c This method tests for transit peptides characteristic of localization to chloroplast, mitochondrion, or ER. The probability of the predicted transit peptide is given. d This method obtains scores for presence of a chloroplast, mitochondrial or ER signal peptide, or for some other cellular localization. The winning prediction is placed in a 'reliability class' (RC) according to the difference between the winning score and the next highest score. The classes, in descending order of reliability, are RC1–RC5. [file 1471-2229-8-120-S5.pdf]

Additional Table 5: Chloroplast Transit Peptide Predictions for Starch Metabolic Proteins

**Sequences**	**iPSORT** [a]	**PCLR** [b]	**Predotar** [c]	**TargetP**	**Consensus prediction**
At4g24620 PGI1	Chloro	Pos(0.992)	Plastid(0.92)	Chloro RC1 [d]	Chloro
At5g51820 PGM1	Mito	Pos(0.825)	Plastid(0.95)	Chloro RC5	Chloro
AT1G70820 PGM2	Mito	Pos(0.993)	Plastid(0.97)	Chloro RC1	Chloro
At5g19220 APL1	Chloro	Pos(0.716)	Plastid(0.60)	Chloro RC1	Chloro
At1g27680 APL2	None	Neg(0.301)	None(0.96)	None RC5	Not chloro
At4g39210 APL3	None	Pos(0.558)	Possibly plastid (0.32)	None RC2	Not chloro
At2g21590 APL4	Chloro	Pos(0.578)	None(0.92)	None RC5	Not chloro
At5g48300 APS1	Chloro	Pos(0.961)	Plastid(0.92)	Chloro RC1	Chloro
At1g05610 APS2	Chloro	Pos(0.662)	None(0.79)	Chloro RC4	Chloro
At1g32900 GBS1	Mito	Pos(0.546)	Plastid(0.92)	Chloro RC1	Chloro
At5g24300 STS1	Mito	Pos(0.881)	Plastid(0.95)	Chloro RC2	Chloro
At3g01180 STS2	Chloro	Pos(0.887)	Plastid(0.95)	Chloro RC2	Chloro
At1g11720 STS3	None	Pos(0.864)	None(0.89)	Chloro RC2	Not chloro
At4g18240 STS4	Mito	Pos(0.572)	Plastid(0.50)	Chloro RC2	Chloro
At3g20440 SBE1	Mito	Pos(0.665)	Possibly plastid (0.33)	Chloro RC4	Chloro
At2g36390 SBE3	None	Pos(0.951)	Plastid(0.63)	Chloro RC2	Chloro
At5g03650 SBE2	Chloro	Pos(0.585)	Possibly mito(0.38)	Chloro RC5	Chloro
At2g39930 ISA1	Mito	Pos(0.601)	None(0.85)	Chloro RC3	Not chloro
At1g03310 ISA2	Chloro	Neg(0.394)	Plastid(0.85)	Chloro RC2	Chloro
At1g69830 AMY3	Chloro	Pos(0.865)	Possibly plastid(0.47)	Chloro RC1	Chloro
At1g10760 GWD1	None	Pos(0.539)	None	Chloro RC5	Not chloro
AT5G26570 GWD3	Chloro	Pos(0.93)	Plastid(0.54)	Chloro RC1	Chloro
At5g64860 DPE1	Chloro	Pos(0.973)	Plastid(0.84)	Chloro RC3	Chloro
At3g29320 PHS1	Chloro	Pos(0.608)	Possibly plastid(0.24)	Chloro RC4	Chloro
At4g09020 ISA3	Chloro	Pos(0.979)	Possibly plastid(0.32)	Chloro RC1	Chloro
At3g23920 BAM1	None	Pos(0.652)	Plastid(0.51)	Chloro RC5	Chloro
At4g00490 BAM2	Mito	Pos(0.909)	Plastid(0.72)	Chloro RC2	Chloro
At4g17090 BAM3	None	Pos(0.883)	Possibly plastid(0.25)	Chloro RC3	Chloro
At5g55700 BAM4	None	Neg(0.286)	None(0.95)	None RC4	Not chloro
At5g04360 LDA1	Chloro	Pos(0.955)	Plastid(0.97)	Chloro RC1	Chloro
At5g17520 MEX1	Mito	Pos(0.677)	Possibly plastid(0.29)	Chloro RC1	Chloro
At5g46110 TPT1	Mito	Pos(0.548)	Mito(0.45)	Mito RC4	Not chloro
At5g16150 GLT1	Mito	Pos(0.473)	Possibly mito(0.30)	Chloro RC5	Not chloro
