# Supplementary figures and images for: Arabidopsis At5g39790 encodes a chloroplast-localized, carbohydrate-binding, coiled-coil domain-containing putative scaffold protein
Source: BMC Plant Biol. 2008 Nov 27;8:120. doi: 10.1186/1471-2229-8-120 (PMC2653042; doi:10.1186/1471-2229-8-120)

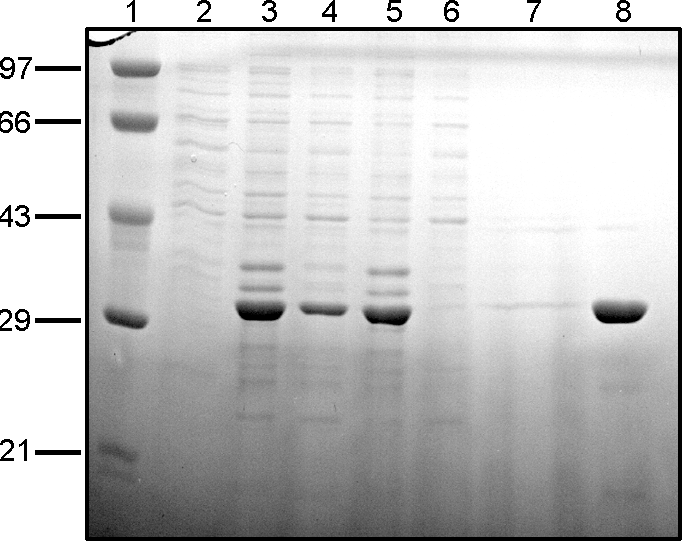

Supplement: Additional file 8 — Supplemental Figure S1. Purification of At5g39790 (-CTP) used for the preparation of antibodies in rabbit. Purification of At5g39790 for antibody preparation: Lane 1, Low MWt standards; lane 2, 3 μg French press supernatant; lane 3, 3 μg of French press pellet (50,000 × g); lane 4, 3 μg of urea supernatant; lane 5, 3 μg of Urea pellet (125,000 × g); lane 6, 3 μL Ni-NTA flow-through; lane 7, 30 μL column wash; lane 8, 3 μg column eluant. [file 1471-2229-8-120-S8.tiff]
